# Influence of sport expertise in facilitating and inhibiting the recognition of the opponent’s intentions in sailing

**DOI:** 10.3389/fnhum.2023.1285963

**Published:** 2024-01-08

**Authors:** Alice Cancer, Chiara Pirola, Leonardo Fogassi, Alessandro Antonietti

**Affiliations:** ^1^Department of Psychology, Università Cattolica del Sacro Cuore, Milan, Italy; ^2^Department of Medicine and Surgery, University of Parma, Parma, Italy

**Keywords:** mirror neuron system, sport, sailing, intention recognition, action anticipation, deception

## Abstract

Starting from the proposed role of the mirror neuron system in the recognition of the intention underlying the actions of others, an experimental paradigm was implemented to test the role of sailing motor expertise in predicting the outcome of a competitor’s action. It was hypothesized that subjects with experience in sailing would correctly interpret the maneuver performed due to the activation of domain specific motor representations of the same movements and that subjects who practiced a sport different from sailing would perform worse because of the activation of irrelevant motor patterns. For doing so, a series of video clips, in which a professional sailor performed a tack or a feint, have been manipulated so that the video clips would stop at the moment of the dunkin, namely, when the boat acquires speed to tack or continue straight ahead. The task consisted in predicting whether the action following the dunkin was an actual tack or a feint. The performance of 87 subjects, divided into three subgroups (sailors, tennis players, sedentary), was evaluated in terms of accuracy in identifying the sailor’s intentions and correlated to age, gender, manual dominance, education, job, hours spent weekly playing videogames, and experience in playing sports. Results showed that the percentage of correct identifications of the intention to do a tack or feint was the highest in the group of sailors and the lowest in tennis players. An inverse relation between tennis experience and ability in recognizing the sailor’s intention was found in the group of tennis players. Gender, age, manual dominance, education, job, and experience with videogames were not found to be correlated with performance. Findings support the possible implication of the mirror neuron system in maneuver detection in sailing and may be a starting point for the development of psychological training in this sport.

## 1 Introduction

The ability to form anticipatory representations of ongoing actions is crucial for effective interactions in dynamic environments. Numerous studies have shown that the perception of actions is closely linked to motor representations (Prinz, 1997; Hommel et al., 2001). According to this view, observed actions are directly matched to the motor representation of these actions. Since the outcome of a motor representation is known, once the motor representation is activated in the observer, this allows the individual to retrieve the meaning of the observed action, thus to understand it directly (Rizzolatti et al., 2001). The neural basis of this direct correspondence mechanism is identified in the mirror neuron system (MNS), a neural network which not only is able to encode the goals of motor acts performed by other individuals, but also action goals, allowing people to recognize the intentions underlying others’ actions (Iacoboni et al., 1999) and others’ emotional states as well (Corradini and Antonietti, 2013).

### 1.1 The MNS and the recognition of others’ intentions

Fogassi et al. (2005) recorded parietal mirror neurons’ activity while a monkey performed an experimental paradigm involving two different situations: in the first, starting from a pre-determined hand starting position, it had to grasp a piece of food placed in front of it and then bring it to its mouth; In the second, it had to grasp the food (or an object) and place it into a container. The results of the experiment showed that, during the grasping act, neurons were activated differently depending on whether the subsequent motor act consisted of bringing to the mouth or putting into the container. This showed that motor neurons can code the final goal of complex action sequences, that is, the motor intention of the agent. A similar specificity was also found when the monkey observed the experimenter performing the same actions, i.e., mirror neurons activated differently depending on the type of observed action. Importantly, both during the monkey actual action execution and during the observation of the experimenter’s action, the neurons were activated when the hand prefigured to take the food. The fact that the visual stimulus of the experimenter grasping the food or another object also activates the same neural pattern, i.e., the set of potential motor acts that precede the animal’s execution not only of that act but of the entire chain, shows that the monkey was able to immediately grasp the intention underlying other’s behavior. This finding led to the interpretation that the differential activation of the MNS allows the observer to predict what the observed agent is going to perform next. As a consequence, also the observer can perform different types of reactions. Both electrophysiological and brain imaging studies demonstrated that also in humans there are mechanisms and circuits, similar to those identified in the monkeys, involved in understanding others’ motor intentions. In human studies it has been shown that the observation of a motor act performed in different contexts, automatically suggesting different actor’s intentions, differentially activate the frontal node of the MNS. For example, in a study by Iacoboni et al. (2005) volunteers were presented with three types of conditions: in the first, they observed the videoclip of a scene containing objects arranged as if someone was about to consume a tea or had just finished doing so (context); In the second, they observed a hand grasping a teacup with a forceful or precise grip (action); In the third, the subjects observed the same grasping action as in the second one, but within one of the context of the first condition, such as to suggest the intention to take the cup to bring it to the mouth and drink or the intention to take it to move it and tidy it up (intention). Comparing the brain activations between the intention condition and the addition of the other two conditions, it appeared a differential activation of the right inferior frontal gyrus, that is part of the MNS. This indicates that this system is able to encode not only the observed act, but also the underlying intention, suggesting that when the observer witnesses the execution of a motor act made by another individual, the activation of the MNS predicts the subsequent acts of the action chain. Notably, this activation allows the observer to automatically understand the meaning of the observed behavior without any reflexive, conceptual, and/or linguistic mediation.

Research about the MNS suggests that understanding the actions of others is allowed by comparing, in the observers, their motor representations constructed with their prior experience and the sensory input provided by observing equivalent actions done by others (motor resonance). Consistently with the motor resonance hypothesis, in one fMRI study, Calvo-Merino et al. (2004) examined differences in the MNS activation of experienced ballet and capoeira dancers (two very similar dance types) during observation of videoclips showing steps of both types of dance. They found that the intensity of activation in action-sensitive brain areas, including the premotor cortex (BA6), was higher when participants observed dance in the style in which they were expert. In a subsequent study by the same research group (Calvo-Merino et al., 2006), brain activations of male and female dancers were compared during observation of gender-specific classical ballet moves. Since female and male dancers usually train together during practice, all dancers were visually familiar with all moves, but they experience motorically only the gender-specific ones. The findings of fMRI activation showed greater cortical and cerebellar activity when dancers viewed moves belonging to their own motor repertoire, compared to opposite-gender moves that they frequently saw but did not perform, suggesting a greater importance of motor expertise in activating the MNS, compared to perceptual expertise. Thus, motor experience plays a significant role in perceptual processing and action prediction. Accordingly, observer’s motor skills (such as the ability to kick or throw accurately) exert a direct effect on the understanding of these actions performed by others (Blakemore and Decety, 2001; Wolpert et al., 2003; Wilson and Knoblich, 2005).

### 1.2 The recognition of others’ intentions in sport

The ability to anticipate what is going to happen and make decisions accordingly is an important component of success in sports (Mann et al., 2007; Müller and Abernethy, 2012; Williams et al., 2018a; Williams and Jackson, 2019). This is relevant because of significant spatial and temporal constraints, which require information to be processed in relatively short time periods in order to allow athletes to plan and execute a timely response to the actions of their opponents (Triolet et al., 2013; Cañal-Bruland and Mann, 2015; Williams et al., 2018b).

One of the first published studies on the importance of anticipation in sport was carried out in tennis using the video-based temporal occlusion method (Jones and Miles, 1978). The authors presented participants with footage of an opponent executing a serve from a first-person perspective. Participants observed each serve and were then asked to anticipate the direction in which the opponent would hit the ball. At various moments relating to racket contact with the ball, the footage was occluded to prevent access to later sources of information. Participants were thus forced to make a judgment based on the information available up to the point of occlusion. The results showed that experienced tennis players were much more accurate in making anticipatory judgments than another group of less experienced players.

This result stimulated numerous investigations in which the video-based temporal occlusion paradigm has been used. The generalizability of findings has been explored across a range of different sports, including cricket (Abernethy and Russell, 1987), field hockey (Starkes, 1987), squash (Abernethy, 1990), volleyball (Wright et al., 1990), soccer (Williams et al., 1993), badminton (Abernethy and Zawi, 2007), and baseball (Müller and Fadde, 2016). Subsequent studies largely confirmed the relevance of being able to detect postural cues when anticipating an opponent’s actions in sport (Abernethy et al., 2008; Aglioti et al., 2008; Urgesi et al., 2012; Tomeo et al., 2013).

Behavioral studies showed that opponents’ body kinematics provide important anticipatory cues (Abernethy and Zawi, 2007; Abernethy et al., 2008). As a result, video-based temporal occlusion protocols have been prominent in fMRI studies of the neural correlates of anticipation. Typically, during an action prediction or anticipation task contrasted with a control condition, experts and novices showed greater activation in the action observation network (AON) when asked to predict an action versus passively observing it (Wright et al., 2011; Abreu et al., 2012; Bishop et al., 2013). Overall, fMRI studies demonstrated that areas of the AON are more strongly activated in sports anticipation tasks than in control tasks in both the experts’ and novices’ brains and that areas showing higher activation in the experts are mainly found in the AON (Yang, 2015; Smith, 2016).

Transcranic magnetic stimulation (TMS) has been included in the temporal occlusion paradigm. Aglioti et al. (2008) asked basketball athletes to predict the outcome of some basket shots. They predicted the outcome sooner and more accurately than observers with relevant visual experience (coaches and sportswriters). TMS applied to the motor cortex revealed facilitation of motor evoked potentials (MEPs), while both groups observed hoop shots from a lateral perspective. However, there was also a very specific facilitation of MEPs that occurred only in visual-motor experts (basketball players) and not in visual-only experts (expert coaches). This effect occurred only when participants observed shots that were not going to land, only in relation to the release point, and only in reference to a hand muscle involved in controlling the ball’s trajectory. This study provided evidence in favor of motor resonance as the basis for anticipation in basket.

Tomeo et al. (2013) presented participants with video sequences of penalty kicks showing both the run-up and shooting action, as well as part of the ball trajectory. Half of the trials were manipulated in such a way that the trajectory of the ball was reversed to mirror shortly after the kick (incongruent condition). Since the body kinematics were not mirror-reversed, the soccer players’ interpretation of the body kinematics would have been to their disadvantage, leading to a higher level of errors in incongruent trials than goalkeepers or beginners who rather relied predominantly on the trajectory of the ball. In a second experiment, TMS was used to probe the cortico-spinal excitability of the lower leg and forearm muscles during the observation of incongruent versus congruent actions. It was found that experienced kickers showed a motor facilitation selectively for the representations of the muscles that are used to perform soccer actions, namely, the leg muscles. This is consistent with a specialized neural representation in kickers due to motor resonance. Along this line of research, Cancer et al. (2022) implemented a similar task to predict the trajectory of the ball in penalty kicks. The task involved the manipulation of a series of penalty kick movies, interrupted at the moment when the kicker’s foot touched the ball, and the subsequent request to identify as quickly as possible the trajectory the ball would take in four possible directions. The results, assessed in terms of accuracy and speed, suggested that when the observers analyzed the kicker’s kinematics (from the penalty kicker’s run-up to the contact of his foot with the ball), they activated their own motor representations of that action, which enabled them to recognize it.

Balser et al. (2014) explored via fMRI whether motor experience activates different patterns depending on the type of anticipation required (spatial or motor). Experienced and novice tennis players watched video clips depicting forehand strokes; Their task was either to indicate the expected direction of the ball trajectory (spatial anticipation) or to decide on an appropriate response to the observed action (motor anticipation). The experts performed better than the beginners in both tasks, but they also showed stronger neural activation in the regions of the AON. In the experts there were no differences between the tasks and this was attributed to their improved motor representations acquired through years of training (i.e., more sophisticated motor resonance).

### 1.3 The recognition of others’ intention in sport in the case of deceiving

In the context of sport, in order to deceive the opponent, extensive use is made of “feints,” which should lead the observer to believe that the athlete’s intention is different from the one he/she will really implement. Ripoll et al. (1995) measured the accuracy of experienced, intermediate, and novice boxers when responding to strikes, openings, and feints. Participants watched six 60-s video sequences, each of which showed a subject simulating attacks, openings, and feints. The participants’ job was to respond by moving a joystick left or right (to avoid an attack), forward (for an opening), or in no direction (for feints). The analysis revealed that experts gave more responses to the feints (47%) than intermediates (30%) and beginners (23%), thus being more susceptible to deceptive actions.

In most of the feint studies, the researchers used tasks where the response options were similar, such as judging whether a player will move left or right. Jackson et al. (2006) tested the ability of experienced and novice rugby players to judge the change of direction when an approaching player moves left, right, or sidesteps deceptively, pretending to move in one direction before moving in the opposite direction. Skilled players were more accurate than novices in judging deceptive “lateral passes” but not when judging non-deceptive actions, suggesting that the ability to anticipate an opponent’s actions extended to discriminating between genuine and deceptive actions. This finding was replicated by Brault et al. (2012).

What motor cues are used to identify feints? Smeeton and Williams (2012) analyzed the kinematics of penalty kicks and found significant differences between non-deceptive and deceptive kicks at multiple markers spanning both the upper and lower body and across all occlusion points. Iwatsuki et al. (2016) reported similar results when they compared normal tennis backhand drop shots or when they gave the impression of setting up a backhand slice. Kinematic analysis revealed that the center of mass (COM) moved more forward, horizontal shoulder rotation and twist angle were greater, and racket surface area was higher on tricky drops than on unmasked drop shots. In a similar vein, Lopes et al. (2014) found that linear combinations of multiple variables were stronger predictors of penalty kick direction than local variables.

Brault et al. (2012) used kinematic analysis to identify the differences between non-deceptive sidesteps and changes of direction in soccer and to determine the characteristics of the most effective and least effective sidesteps. In a follow-up study, Brault et al. (2012) showed that experts were more attuned to the “honest” COM shift signal, while non-gamers were more attuned to deceptive signals. Thus, these results provide tentative evidence that the advantage held by high-skilled players over low-skilled players may lie in their greater sensitivity to global information.

To determine the contributions of perceptual and motor skills in judging deceptive actions, Cañal-Bruland and Schmidt (2009) compared the ability of experienced goalkeepers, experienced players, and novice players to differentiate genuine (ball released) and false (ball held) penalty shots in handball. They found that the results of goalkeepers and experienced players did not differ, as both groups were better than novices at discriminating between authentic and deceptive shots. The authors noted that the advantage of experienced goalkeepers and players over novices cannot be attributed solely to motor experience. Nonetheless, the fact that expert players behaved like goalkeepers is consistent with the assumption that motor experience facilitates task execution (Cañal-Bruland et al., 2010).

### 1.4 Recognition of others’ intentions in sailing

One sport in which the ability to anticipate an event before it occurs and to make an effective decision is crucial is sailing. Sailing requires a high level of perception of stimuli and at the same time an excellent ability to extract significant information from the environment, anticipate the appearance of the main visual indicators, and make decisions that facilitate continuous adaptation to contextual constraints. The ability to anticipate an event before it occurs and to take an effective decision is critical for sailors because weather conditions and the actions of competitors change and are unstable (Araújo et al., 2005, 2015). Given the dynamism of the context, the way of navigation and the behavior of the sailors must be continuously adapted.

From an analysis of the literature, there are no studies that investigated anticipatory representations and the understanding of the opponents’ intentions in sailors.

### 1.5 Purposes of the present study

The aim of the present study was to investigate the processes involved in identifying the intentions of an opposing sailor during a simulated regatta, based on the expected involvement of the MNS. It is expected that sailors, due to their experience and practice in the domain, perform better in recognizing the intentions underlying the maneuver they see to be performed by their opponents than those who do not participate in any sport (sedentary). It is also hypothesized that expertise in a sport other than sailing will produce worse results, even compared to sedentary people, because the behavior observed in the sailor elicits motor patterns which are relevant in the domain of the sport they practice but not in sailing, likely inducing the inhibition of the correct motor representations. Tennis was chosen as the comparison sport because, as in sailing, the opponent is in front of the athlete and can change his/her position in space and uses the whole body to perform the gestures typical of that sport. Tennis also has characteristics of dynamism and speed as in sailing.

Another aim of the study is to investigate whether gender, age, hand dominance, education, job, and game experience have an influence on the task of identifying the opponent’s intentions. Videogame experience was assessed to control its possible influence on task performance, which might have some features (e.g., fast responding, identifying deceiving behaviors, and so on) similar to videogames. In fact, Gold and Ciorciari (2021) analyzed the performance of experienced and novice video game participants in an action occlusion task in football: The results showed that expert participants, due to the mental representations developed in videogame practice, performed better than novices despite the fact that both were not experts in that sport.

Finally, by means of a self-report questionnaire, we aimed at understanding at which extent individuals are aware of the process through which they try to grasp sailor’s intentions, their perception of the mechanisms involved in the detection of the maneuver and what clues, coming from the sailor’s body and/or the boat, participants rely on to identify sailor’s intentions. Their beliefs about the nature of the skills involved in intention recognition were investigated as well.

## 2 Materials and methods

### 2.1 Sample

Ninety subjects aged between 18 and 66 years (*M* = 31.8, SD = 13.4) participated in the study. They were divided into three groups: sailing (*N* = 30), tennis (*N* = 30), and sedentary (*N* = 30). In the tennis group, three participants were excluded from the sample as they did not respond to all stimuli proposed in the experimental task.

The participants were recruited via advertisements on sport websites according to a non-probabilistic reasoned choice sampling. The inclusion criteria were to be 18 years or more and practicing sailing, tennis, or no sport. Specifically, sedentary people should report to play any sport or train for less than 2 h per week, while sportsmen were required to have at least 1 year of experience.

### 2.2 Experimental task

Participants were asked to observe a series of videotaped sailing actions performed by a professional sailor while he was about to make or not a tack (that is, letting the boat change direction by receiving the push of the wind from the opposite side). The videos were edited so that the footage was interrupted immediately before the main action (namely, during the dunking phase, when the boat gains speed in order to tack or to continue straight ahead by performing a feint), using the temporal occlusion method (Jones and Miles, 1978). Subjects were asked to judge, as quickly as possible, whether the sailor was making a tack or a feint (namely, the boat, despite the preliminary actions similar to those performed to make a tack, would have continued to proceed in the same direction as before).

The videos were created in collaboration with the “Club del Mare” [Sea Club] in Diano Marina, Italy. A professional sailor was asked to perform a series of turns and feints, for a total of 100 videos, 42 of which were selected based on criteria such as framing and video quality. To avoid distracting elements, the maneuvers were all performed in the same way, always using the same tacking style (tacking with roll) at an average speed. Furthermore, the sailor was asked to avoid distracting movements and facial expressions (e.g., looking at the camera or smiling).

**FIGURE 1:**
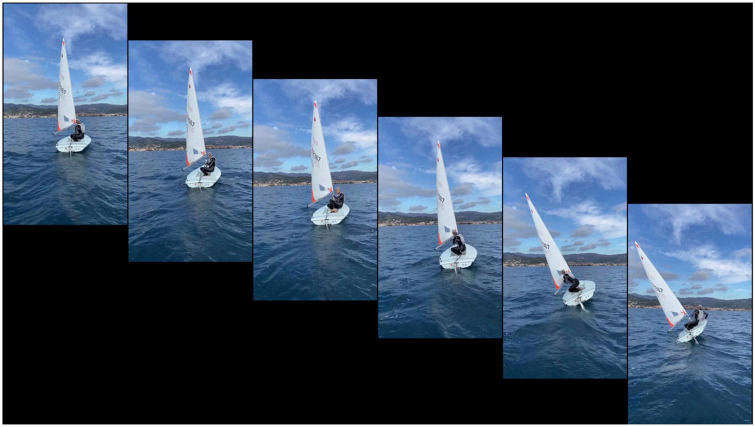
Frame sequence extracted from a videoclip showing the sailor performing a turn.

The videos were taken in appropriate weather conditions, namely, calm sea and suitable light to make movements visible and distinguishable. The used boat was a Laser. The footage was shot using an iPhone 12 (30 fps) from a boat following the sailor, a perspective that simulated a regatta and thus the view of an opponent. Figure 1 shows the frame sequence extracted from the videoclip of a turn trial.

The task was administered thorough an online experiment administration platform (Gorilla experiment builder).^1^ Following two practice trials, each participant was shown 40 video clips (20 turns and 20 feints), presented in full-screen in a randomized order. Before each video clip, participants observed a fixation cross for 1,000 ms. After watching each video clip, participants had a maximum of 5,000 ms to respond (i.e., press a “tuck” or “feint” button), otherwise a missing response was recorded.

### 2.3 Pre- and post-test evaluation questionnaires

Before and after the execution of the task, two questionnaires were administered to the participants.

#### 2.3.1 Pre-task questionnaire

The questionnaire was constructed *ad hoc* and consisted of two parts. In the first one, the participant’s data (gender, age, dominant hand, educational qualification, professional occupation/field of study) and the weekly hours spent by them in front of video games were collected. Then, the respondent was asked whether they played sailing, tennis, or no sport. Depending on the answer, participants who played one of the two sports were given a specific set of questions to investigate their experience. For sailing, these variables were assessed: years of practice, type of boat used, experience on a boat with two athletes, role on the boat, number of training sessions per week, participation in regattas (national, European, world, or Olympic). For tennis, the investigated variables were: years of practice, number of training sessions per week, participation in tournaments and their level.

#### 2.3.2 Post-task questionnaire

It was administered with the aim of assessing the subjects’ perception of the task. Specifically, participants were asked, via an open-ended question, to recount (1) the process that led them to identify whether or not the sailor was turning. Subsequently, through a closed-ended question (yes/no), (2) their perception regarding the correctness of the answers and (3) their opinion regarding the possibility that a different perspective would have facilitated the understanding of the maneuver were investigated. Next, through a multiple-choice question it was asked whether (4) they believed that the ability to detect the sailor’s intentions can improve as a result of training or is an innate ability. Finally, it was investigated whether (5) participants had looked at one or more of the sailor’s body parts (leg, foot, torso, arm, other) or the boat (rudder, sheet, bow, other) during the task.

### 2.4 Procedure

The task was carried out by all participants remotely, using their PCs at home. Subjects who wanted to participate in the study received an e-mail that contained the link to access the Gorilla platform. In the e-mail invitation, subjects were also asked to perform the task in a quiet place, free from any distractions.

Upon opening the link, before the start of the experiment, participants were presented with an informed consent. Upon accepting, they were asked to complete the pre-task questionnaire, the experimental task (average completion time = 10 min), and the post-task questionnaire.

## 3 Results

### 3.1 Group differences

To check whether the three groups (i.e., sailors, tennis players, sedentary) were matched for individual characteristics, group comparisons were tested by means of the chi-squared test for categorical variables and *t* test or one-way ANOVA for continuous variables.

Considering the complete sample, gender was evenly distributed (41 f; 46 m). Gender differences between groups were not significant, χ^2^ = 1.79, *p* = 0.410. Age differences between groups was significant, *F*(2, 84) = 5.20, *p* = 0.007: *Post hoc* comparisons showed that the significant difference was between the sedentary (*M* = 37.8 years, SD = 16.00) and the sailors (*M* = 27.5 years, SD = 10.90), but neither of them differed from the tennis players (*M* = 30.0 years, SD = 10.05) (sedentary vs. sailors: *p*_*Bonferroni*_ = 0.008; sedentary vs. tennis players: *p*_*Bonferroni*_ = 0.074; sailors vs. tennis players: *p*_*Bonferroni*_ = 1.0).

With regard to handedness, 79.3% of the subjects were right-handed, 18.4% left-handed, and 2.3% were not completely lateralized. As for the education level, 11.5% had a secondary school diploma, 48.3% a high school diploma, 25.3% a bachelor’s degree, 13.8% a master’s degree, and 1.1% a doctorate. Students were 41% of the sample, while 59% were workers. Chi-squared test confirmed that groups did not differ for handedness, χ^2^ = 6.33, *p* = 0.18, education, χ^2^ = 4.37, *p* = 0.63, and job, χ^2^ = 2.77, *p* = 0.75.

Concerning the time spent playing video games, the participants reported a time range from 0 to 20 h per week (*M* = 1.52, SD = 3.09), with no significant group differences [Sedentary participants: *M* = 1.37, SD = 2.53; Tennis players: *M* = 1.52, SD = 2.98; Sailors: *M* = 1.67, SD = 3.74; *F*(2, 84) = 0.07; *p* = 0.93].

As for the groups who practiced sports (i.e., sailors and tennis players), the sailors had been sailing on average for 11 years (*M* = 11.60, SD = 9.23). All subjects reported having been on a double at least once: 55.2% as helmsman and 44.8% as bowman. As far as training is concerned, 41.4% said they trained once a week, 34.5% twice, 13.8% three times, 6.9% four times, while 3.4% said they had training sessions during all the week. The majority of the sample of sailors (96.7%) participated in regattas: 46.5% in national championships, 26.7% in European championships, 20% in world championships, and 6.7% in the Olympics.

As for the group of tennis players, they have been playing tennis on average for 8 years (*M* = 8.38, SD = 9.63). 41.4% of them claimed to train once a week, 34.5% twice, 13.8% three times, and 6.9% four times. Half of the sample participated in tournaments: 75% as amateurs and 25% as professionals.

Years of practice comparison between the tennis and sailing groups revealed that there was no significant difference [*t*(55) = 1.13, *p* = 0.26]. Even the number of practice session per week did not differ between sailors and tennis players [*t*(55) = 0.05, *p* = 0.96].

### 3.2 Effect of sport experience on the experimental task

**FIGURE 2:**
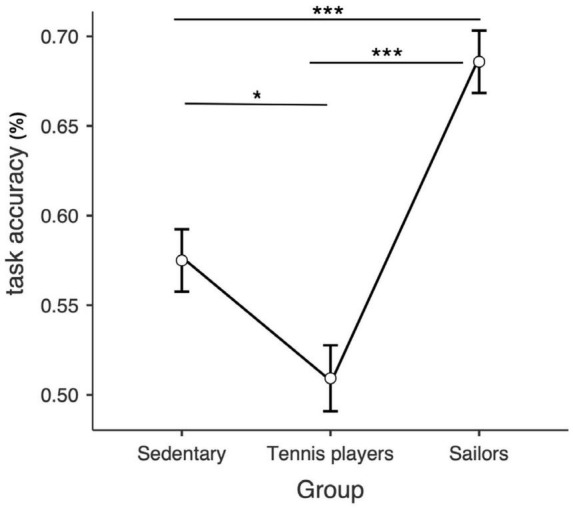
Estimated marginal means of the detection task accuracy for each group. ***p < 0.001; *p < 0.05.

Group comparison was performed by means of a one-way ANOVA. The *a priori* power analysis revealed that a sample size *n* = 90 was enough for detecting a medium effect size (η^2^ = 0.10) with a power of 0.84 and alpha set at 0.05. The group comparison in the experimental task performance showed a significant difference in the detection accuracy rate—measured as the percentage of correct responses on the complete set of stimuli—*F*(2, 85) = 25.1, *p* < 0.001, η^2^ = 0.37. All *post hoc* pairwise comparisons (using Bonferroni correction) were significant (*p*-values ranging from < 0.001 to 0.03), with sailors being the most accurate and tennis players the least accurate (Figure 2).

The correlation between years of practice and task accuracy (considering the tennis and sailing subsamples only) was non-significant (*r* = 0.04, *p* = 0.75). Considering the sailors only, no correlation between years of practice and task accuracy emerged (*r* = 0.044, *p* = 0.82). On the other hand, years of practice and task accuracy correlated negatively in tennis players (*r* = −0.38, *p* = 0.05).

To investigate whether it was easier for the participants to detect if the maneuver was an actual tack or a feint, a 2 × 3 mixed factorial ANOVA was conducted. The *a priori* power analysis revealed that a sample size of *n* = 90 was enough to detect a medium effect size (η^2^ = 0.10) with a power of 0.99 and alpha set at 0.05. To exclude a potential response bias, we used the Signal Detection Theory approach (Macmillan and Creelman, 2004) to calculate a criterion score representing the bias toward the tack response, as follows: *C* = −[z(Hit) + z(FA)]/2, where Hit represents the number of correctly identified tacks and FA represents the number of feint items which were identified as tacks. Given that the average bias was very close to 0 (*M* = 0.00; SD = 0.62), we concluded that the decisions of our participants were unbiased. Furthermore, the criterion score group comparison was non-significant [*F*(2, 84) = 0.26; *p* = 0.77].

**FIGURE 3:**
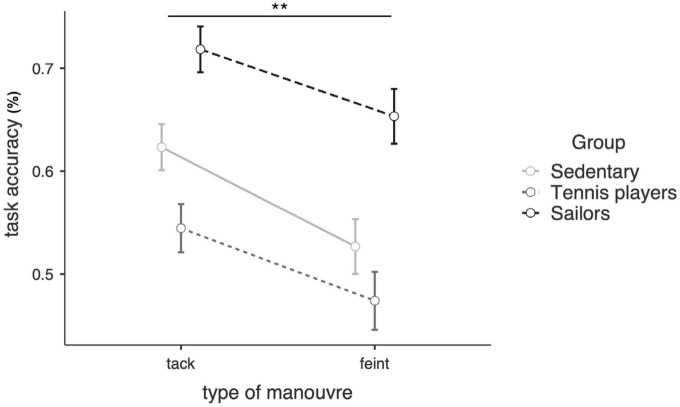
Estimated marginal means of the detection task accuracy for tack and feint separately. **p < 0.01.

The effect of the type of maneuver (tack vs. feint) was statistically significant, *F*(1, 86) = 14.5, *p* < 0.001, η^2^ = 0.06, whereas no significant interaction effect emerged, *F*(2, 84) = 0.24, *p* = 0.79. Results revealed that, regardless of sport experience, it was easier to detect tacks rather than feints (Figure 3).

### 3.3 Post-test questionnaire

The self-report answers on decision making (“Would you be able to describe the process that led you to identify whether the sailor made the turn or not?”) revealed that 75% of the subjects stated that they were unable to explain how they identified whether it was a feint or a turn. In addition, 59.8% of participants reported that they thought they answered incorrectly most of the times. As regards to the question on point of view change (“Do you think that watching the videos from another perspective would make it easier to understand the maneuver?”) most subjects (59.6%) claimed that changing the perspective of the videos would not help them anticipate the sailor’s intentions. When asked about training or an innate skill (“Do you think that in order to detect whether it is a tack or not, it is more important to train or to have an innate skill?”), almost all participants (97.8%) reported that it was important to train in order to be able to understand the maneuver rather than to have an innate skill.

As the observed body parts of the sailor in the videos (“To detect whether or not the sailor makes the turn, did you observe a body part of him? If yes, which one?”), 96.6% said they observed a body part to detect whether the sailor was making the turn. More specifically, the most commonly observed parts were torso (57%), arm (26%), foot (22%), and leg (18%). Analyzing each group separately, comparison between subgroups showed that tennis players focused more frequently on the arm (55.2%), χ^2^ = 15.1, *p* < 0.001, and sailors focused more frequently on the leg (40%) χ^2^ = 11.9, *p* = 0.003, and the foot (50%), χ^2^ = 15.4, *p* < 0.001, than the other groups did; The torso was observed equally by all three groups. Furthermore, participants who reported to have observed the foot were significantly more accurate in the detection task [*t*(85) = −2.99, *p* = 0.004, η^2^ = 0.09].

When asked about the observed part of the boat (“To detect whether or not the sailor turns, did you observe a particular part of the boat? If yes, which one?”), most subjects (77.5%) reported that they looked at a part of the boat before deciding whether it was a tack or a feint. The most frequently observed parts of the boat were bow (36%), rudder (29%), and sail (22%). No significant differences were found in these responses among groups, χ^2^ = 8.20, *p* = 0.08.

## 4 Discussion

### 4.1 MNS and recognition of opponent’s intentions

Research on the MNS suggests that understanding the intentions of others’ actions is enabled by motor resonance, i.e., the coupling between motor representations of actions constructed on the basis of the observer’s previous experience and the sensory input provided by observing the equivalent actions performed by others. Specifically, the motor resonance matching mechanism assumes that during the observation of an action, the chain of motor acts representing that specific action is activated in the motor repertoire of the observer (Fogassi et al., 2005; Chersi et al., 2011), allowing her/him to recognize the underlying intention. Although no neurophysiological data were collected in the current study, the behavioral results obtained by the sailors suggest the possible implication of the MNS in identifying the maneuver (tack or feint) performed by another athlete. The motor experience of the sailors, and in particular the activation of corresponding motor representations, played a significant role in the prediction of the sailor’s action, allowing them to recognize, in a higher percentage than the other groups, the intentions of the observed agent, i.e., if he wanted to make a real turn or make a feint.

Interestingly, tennis players performed the worst in this paradigm. It is likely that, due to their developed practice in their own sport domain, they were more sensitive to sources of information close to the final effector, such as the arm or racket (Huys et al., 2009). In fact, when observing an action sequence, athletes tend to direct their gaze toward the segments of the body that initiate the movement and then progressively move their gaze toward the next segments. Decisions are then processed in a proximal to distal fashion following gaze exploration. The answers to the post-test questionnaire confirmed that tennis players, to determine whether it was a feint or a tack, mainly focused on the sailor’s arm, not taking into consideration other fundamental body parts (such as torso and feet).

As a measure of action prediction, we have only considered tack/feint detection accuracy. It would have been interesting to study differences in response times as well. However, given the online administration of the experiment, we could not consider time measures consistent and reliable, since participants used different devices and operating systems, each of them with slightly different time latencies between the presentation of the stimuli and the registration of the response, to run the task.

Nonetheless, we recognize that a limitation of our study is that we did not control for participants’ perceptual experience as observers in alternative sports than the one they practiced. Previous findings on the role of perceptual vs. motor experience are mixed. Calvo-Merino et al. (2006) found greater brain responses in both female and male dancers watching gender-specific ballet moves, for which they had motor experience. Cañal-Bruland and Schmidt (2009) reported no difference between goalkeepers and players, thus suggesting comparable performance of motor and perceptual experts. Instead, results from Tomeo et al. (2013) showed worse performance of kickers vs. goalkeepers, pointing to a greater role of perceptual expertise in the latter group. Given the relevant role of perceptual expertise in action anticipation, future studies should replicate our paradigm considering perceptual experts performances as well. Although we assumed that our participants’ perceptual experience was correlated with their practical sport experience, information on their potential perceptual experience in other domains would further the understanding of the impact of motor experience on action anticipation in sailing, by controlling for the impact of mere perception.

### 4.2 Turns vs. feints

The analysis of the accuracy of the responses for turns and feints showed that for all three groups it was easier to identify when it was a turn rather than a feint. According to the motor resonance hypothesis, the identification of the specific maneuver is based on the recognition of specific kinematic features of the sailor’s body. In fact, it has been suggested that the MNS, in particular its dorsal component, may have an important role in decoding kinematics of the observed action (see Errante and Fogassi, 2019). We can argue that this is due to the fact that the turn and the feint are very similar maneuvers. In fact, it often happens that when a sailor does not need to tack and intends to go straight (feinting), he/she perfectly simulates the movements needed to make a tack, simply emphasizing less the final phase of the luff (the maneuver consisting in turning the boat toward the direction of the wind, so that the angle of incidence of the wind on the sail decreases). The tack requires a complete luff, which brings the boat to be parallel to the wind direction, i.e., with an angle of incidence equal to zero, and then, having rotated the sail above the sailor’s head, produces the progressive increase of the angle of incidence of the wind on the sail on the opposite side of the boat. The luff, in fact, is a peculiarity of the tack which makes it easier to distinguish it from the feint. However, some subjects, seeing the sailor luffing, mistakenly thought he was performing a tack. In fact, when a feint is made, the luff, which is not as accentuated as in an actual tack, is performed not to change direction but to decrease the angle of incidence of the wind on the sail and to accelerate the speed of the boat.

While the sailors also responded correctly to more tack videos, they were still more accurate than the other groups in identifying faints. Several studies have shown that more experienced athletes are more accurate in predicting what will happen next by identifying the options relevant to the task while ignoring the irrelevant ones (Williams and Jackson, 2019). The sailors, in fact, were able to continuously update their expectations and integrate the information relating to the action trends of the observed sailor. With experience, skilled sportsmen learn to discriminate between deceptive and non-deceptive intentions. The skillfulness of experts in judging deceptive actions is based both on the ability to read the dynamic signals of the body and on the ability to integrate kinematic information with contextual information, a fundamental characteristic in a sport such as sailing where the environment is constantly changing.

### 4.3 The role of individual characteristics

We checked whether the years of experience in sports had influenced the performance of the subjects. The results showed that in the group of sailors there was no correlation between experience and the performance. This was probably due to the fact that within the sample there was little differentiation in expertise (all sailors participated to regattas, whereas only half of tennis player participated to tournaments). As regards the group of tennis players, however, an inverse correlation emerged whereby the less experienced subjects obtained the best results, presumably since expert subjects had developed domain specific motor representations that led them to focus mainly on the sailor’s arm.

Variables such as gender, age, motor dominance, education, job, and weekly hours spent playing video games were not associated with performance on the experimental task, although age across groups was not evenly distributed. These findings suggest that individual differences other than sport expertise play a minor role in modulating the ability to detect the opponent’s intentions in sailing.

### 4.4 Retrospective judgments and beliefs

As regards participants’ opinions on the parts of the body and of the boat considered most informative, it emerged that in general the most observed parts of the body were the torso and the arm, while, as far as the boat was concerned, the bow and rudder. It emerged that in general the sailors, compared to the other groups, focused more on the relevant kinematic information (leg and foot) before making a decision. Sailors reported to have looked at the boat at a larger extent than the other two groups. Looking at the boat, especially the bow and the rudder, to understand the action that an opponent is carrying out is very important in sailing because it is precisely the boat which, maneuvered by the sailor, carries out the movement. The results to the questions of the post-test questionnaire underlined the differences between participants skilled in sports practice and naïve subjects: The former mainly focused on the bow, the rudder, the torso, and the foot, while the latter on less informative parts of the body such as the arm and minimally also on the boat. In general, no part of the body or of the boat is informative to understand the maneuver if considered alone. Indeed, sailing is a situational sport, in which it is essential to consider both the information of the body and that of the boat and the surrounding environment (weather conditions, sea, opponents, position of the buoys).

The perspective used in the observation paradigm was found to be functional for the purposes of the task, as it perfectly simulated the point of view of an opponent during a regatta. In fact, the subjects reported that a hypothetical change of perspective would not have helped.

Participants also believed that in order to be successful in the task, it is not necessary to have an innate ability, while training is much more important. For successful anticipations and decisions, at the basis of competence in sport and in other domains, in addition to motor resonance, it can also be important to take into consideration the ability to detect familiarity and recognize stimuli (Williams and Davids, 1995; Abernethy et al., 2005; North et al., 2017). The skillfulness of the sportsmen is guided by a perceptual/cognitive ability that they acquired as a function of extensive sporting practice. As a result of investing so many hours of practice, these individuals developed specific complex memory structures that allow them to have superior perceptual and cognitive processing in encoding, storing, and retrieving significant locations.

Finally, the majority of participants stated that they were not able to describe the process that led them to identify the subsequent behavior of the sailor in the video. This outcome can be analyzed in the context of the implicit/explicit nature of the process under investigation. Behavioral measures in this study were meant at revealing the immediate, implicit, not fully conscious, not introspectable, not verbally expressed understanding of other people’s intentions. Since the rates of correct responses were above the chance level (apart the tennis condition), we can maintain that such understanding occurred in responders. Retrospective questions were aimed at checking whether the implicit understanding was also coupled to an explicit awareness of the ongoing process implicated in trying to catch others’ intentions. The dissociation between implicit and explicit data supports the claim that only implicit understanding occurred.

## 5 Conclusion

The present study showed that sailors’ perception of other people’s intentions is closely linked to their domain specific motor expertise. Numerous behavioral and neurophysiological studies have shown that experts in a particular motor skill are quicker in recognizing, by observing it, the underlying kinematics of that skill and has a greater activation of the motor cortex, compared to subjects less expert in sports (Calvo-Merino et al., 2004). These data are in line with the results here obtained in sailors, who have proved to be much more accurate in recognizing the maneuver than sedentary and tennis players. Effective interactions in dynamic environments such as sailing, in fact, require the prediction of the outcome of the observed actions and the formation of anticipatory representations of movement sequences. The tennis players, on the other hand, obtained the worst results. It would be useful in the future to compare the performance of sailors with those of other individuals who practice a water sport (e.g., windsurfing). In fact, sailing is a sport that differs greatly from those previously studied in sport psychology. Carried out in an unusual environment and normally without contact with the public, the practice of sailing requires the athlete to have knowledge of hydrodynamics, meteorology, navigation conditions, and anticipation of events.

Another suggestion for future research is to show participants a video explaining what a tackle and a feint are before the experimental task begins. Unlike sports like soccer or tennis, where almost everyone knows what a penalty or a serve is, sailing is a less familiar sport.

The data acquired with this paradigm can serve as a basis for the analysis, from a psychological point of view, of the dynamics that occur during a regatta. Furthermore, it would be interesting to test the correlation between this mechanism and the concentration of the sportsmen. During a day of regatta, in fact, it is possible to have from one to three races. The time between them can be short or, depending on weather conditions, very long, to the point where athletes spend hours in the water waiting for the race to start. Concentration and attention are therefore fundamental factors for obtaining optimal athletic performance. Future studies should analyze these variables and also include neurophysiological measures to directly confirm the involvement of the MNS.

### 5.1 Practical implications

Findings from the present study suggest the possible involvement of the MNS in anticipation of sport actions, with prior matching motor experience increasing the performance in early intention detection and alternative sport motor experience decreasing it. Based on these results, it would be possible to implement training interventions to help sailors achieve better psychological preparation with a consequent competitive advantage over their opponents. In line with the findings of the present study, a specific part of these training programs might concern the identification of other sailors’ intentions during a regatta. Exercises based on prediction of the opponents’ behavior, in which the trainees are presented videos like those employed in the experiment described below, might be effective in improving the skill in question. To make these exercises more impacting, the trainees might be instructed to drive the attention toward the parts of the sailor’s body and of the boat which are more informative. According to the MNS mechanism, however, the core of this approach should be the link with personal motor experience. Hence, in the training program, the trainee should be prompted to simulate with her/his body what the opponent is doing on his/her boat. Virtual reality applications might be created to support these activities and markers or sensors might be placed on the relevant parts of the body of the trainee in order to trace her/his movements, allowing the trainer to check if they match the actions performed by the to-be-simulated agent in the virtual environment.

## Data availability statement

The raw data supporting the conclusions of this article will be made available by the authors upon request, without undue reservation.

## Ethics statement

The studies involving humans were approved by the Ethics Committee of the Catholic University of the Sacred Heart in Milan, Italy. The studies were conducted in accordance with the local legislation and institutional requirements. The participants provided their written informed consent to participate in this study.

## Author contributions

AC: Conceptualization, Data curation, Formal analysis, Investigation, Methodology, Supervision, Writing – original draft, Writing – review & editing. CP: Data curation, Investigation, Writing – original draft. LF: Conceptualization, Writing – original draft, Writing – review & editing. AA: Conceptualization, Supervision, Writing – original draft, Writing – review & editing.

## Footnotes

1
https://gorilla.sc
